# Residual C-peptide secretion and hypoglycemia awareness in people with type 1 diabetes

**DOI:** 10.1136/bmjdrc-2021-002288

**Published:** 2021-09-15

**Authors:** Martine J Wellens, Charlotte E Vollenbrock, Pim Dekker, Lianne S M Boesten, Petronella H Geelhoed-Duijvestijn, Martine M C de Vries-Velraeds, Giesje Nefs, Bruce H R Wolffenbuttel, Henk-Jan Aanstoot, Peter R van Dijk

**Affiliations:** 1 Department of Endocrinology, University of Groningen, University Medical Center Groningen, Groningen, Netherlands; 2 Diabeter, Center for Paediatric and Adolescent Diabetes Care and Research, Rotterdam, The Netherlands; 3 Department of Clinical Chemistry, IJsselland Hospital, Capelle aan den IJssel, Netherlands; 4 Department of Internal Medicine, Medical Centre Haaglanden, Den Haag, The Netherlands

**Keywords:** awareness, C-peptide, hypoglycemia, age of onset

## Abstract

**Introduction:**

This study aimed to assess the association between fasting serum C-peptide levels and the presence of impaired awareness of hypoglycemia (IAH) in people with type 1 diabetes.

**Research design and methods:**

We performed a cross-sectional study among 509 individuals with type 1 diabetes (diabetes duration 5–65 years). Extensive clinical data and fasting serum C-peptide concentrations were collected and related to the presence or absence of IAH, which was evaluated using the validated Dutch version of the Clarke questionnaire. A multivariable logistic regression model was constructed to investigate the association of C-peptide and other clinical variables with IAH.

**Results:**

In 129 (25%) individuals, residual C-peptide secretion was detected, while 75 (15%) individuals reported IAH. The median (IQR) C-peptide concentration among all participants was 0.0 (0.0–3.9) pmol/L. The prevalence of severe hypoglycemia was lower in people with demonstrable C-peptide versus those with absent C-peptide (30% vs 41%, p=0.025). Individuals with IAH were older, had longer diabetes duration, more frequently had macrovascular and microvascular complications, and more often used antihypertensive drugs, antiplatelet agents and cholesterol-lowering medication. There was a strong association between IAH and having a severe hypoglycemia in the preceding year. In multivariable regression analysis, residual C-peptide, either continuously or dichotomous, was associated with lower prevalence of IAH (p=0.040–0.042), while age at diabetes onset (p=0.001), presence of microvascular complications (p=0.003) and body mass index (BMI) (p=0.003) were also independently associated with the presence of IAH.

**Conclusions:**

Higher BMI, the presence of microvascular complications and higher age at diabetes onset were independent risk factors for IAH in people with type 1 diabetes, while residual C-peptide secretion was associated with lower risk of this complication.

**Significance of this study**

**What is already known about this subject?**

- Impaired awareness of hypoglycemia (IAH) may develop with longer duration of diabetes.
- Previous studies have reported an association between preserved C-peptide secretion and lower risk of (severe) hypoglycemia.

**What are the new findings?**

- Higher body mass index, the presence of microvascular complications and higher age at diabetes onset were independent risk factors for IAH in people with type 1 diabetes.
- There was a strong association between IAH and having a severe hypoglycemia in the preceding year.
- Residual C-peptide secretion was protective, both for IAH and for severe hypoglycemia (multivariable model).

**How might these results change the focus of research or clinical practice?**

- Even low residual C-peptide secretion has beneficial clinical effect in people with type 1 diabetes.

## Introduction

Impaired awareness of hypoglycemia (IAH) is a serious consequence of long-standing diabetes mellitus. It is defined as the inability to detect the onset of hypoglycemia^1 2^ and thereby the perception of hypoglycemia is attenuated or even absent. IAH develops in approximately 20%–40% of individuals with type 1 diabetes mellitus and 15%–20% of insulin-treated individuals with type 2 diabetes, and lower intensity of symptoms and higher prevalence of IAH increase with longer duration of diabetes.^3–5^ Earlier studies showed that IAH is associated with an increased risk of asymptomatic and severe hypoglycemia^6–9^ and has a strong negative impact on quality of life.^2 10^ Furthermore, cognitive impairment, seizure, coma and even death may occur.^11–13^

Residual C-peptide secretion can be found in approximately 30%–80% of people with long-term type 1 diabetes.^14^ Previous studies have shown an association between preserved C-peptide secretion and the glucagon response to hypoglycemia,^15^ as well as a reduced frequency of self-reported hypoglycemic episodes.^16–20^ Individuals with type 1 diabetes who lacked C-peptide secretion had a fourfold increased risk of severe hypoglycemia.^21^ As the presence of C-peptide was associated with a lower rate of hypoglycemia, it can be hypothesized that residual C-peptide secretion may also be associated with the absence of IAH. An earlier study by Holstein *et al*, in a cohort of 217 individuals with type 1 diabetes, showed that longer diabetes duration, C-peptide status and lower glycated hemoglobin (HbA_1c_) were risk factors for the presence of impaired hypoglycemia awareness.^22^ The aim of the present study was to evaluate to which extent C-peptide and other clinical variables are related to the presence of IAH in a large cohort of individuals with type 1 diabetes.

## Research design and methods

### Study design

The current study is a cross-sectional analysis of data derived from the ‘Biomarkers of heterogeneity in type 1 diabetes’ project, a collaboration between Diabeter, Haaglanden Medical Center (HMC) and the University Medical Center Groningen (UMCG). For the present study, we used baseline data collected between January 2016 and May 2019. The study was approved by the Medical Ethical Review Committee of the UMCG (METC 2015/493). All participants provided written informed consent.

### Study population

Individuals with type 1 diabetes were included between June 2016 and March 2019. Inclusion criteria were a clinical diagnosis of type 1 diabetes, a diabetes duration ≥5 years and age ≥16 years. Presence or absence of IAH was not a criterion for participation. In total, this prospective cohort study included individuals with type 1 diabetes. The baseline evaluation comprised the collection of diabetes-related complaints, comorbidity and complications, medical treatment, laboratory measurements and a dedicated questionnaire. Additional data were collected from the electronic charts and included demographics (age, gender, age at onset, duration of diabetes), physical measurements (height, weight, systolic and diastolic blood pressure, body mass index (BMI)), laboratory data, clinical data (mode of insulin administration, including continuous subcutaneous insulin infusion (CSII)), glucose measurements (including intermittently scanned or continuous glucose monitoring), medication use, alcohol intake, and data on the presence of microvascular and macrovascular complications.

Microvascular complications included microalbuminuria, macroalbuminuria, retinopathy and neuropathy. Microalbuminuria was defined as 20–200 mg/L albumin or an albumin/creatinine ratio between 3.5 and 35 mg/mmol in women and between 2.5 and 25 mg/mmol in men. Macroalbuminuria was defined as >200 mg/L albumin or an albumin/creatinine ratio >35 mg/mmol in women and >25 mg/mmol in men. Retinopathy was scored by an ophthalmologist and was defined according to national guidelines. Self-reported neuropathy scores were obtained from the Diabetes Neuropathy DN4 questionnaire.^23^ The presence of microvascular complications was evaluated using a window of 1 year before and after the baseline visit. Presence of macrovascular complications was defined as (a history of) heart failure, transient ischemic attack or cerebrovascular accident, presence of angina pectoris or myocardial infarction, a previous coronary interventions (either coronary artery bypass grafting or percutaneous transluminal coronary angioplasty).

For the current study, the presence of IAH was assessed using the validated Dutch version of the Clarke questionnaire. This questionnaire comprises five questions on symptoms and frequency of hypoglycemia: each positive answer scored one point. A total score of three or more indicates IAH.^24 25^ Severe hypoglycemia was defined by a positive answer to one of both questions regarding severe hypoglycemia.^25^

### Laboratory measurements

Blood samples for serum C-peptide measurements were collected in the fasting state, between 8:00 and 10:00 in the morning in 482 of the participants, while 27 only had a light breakfast snack. C-peptide concentrations in the latter group were similar to those who were fasting. Routine laboratory data including HbA_1c_, lipid profile, serum creatinine and urinary albumin excretion were either collected on the same day, or obtained from medical records within the last year. Estimated glomerular filtration rate (eGFR) was calculated as described earlier.^26^ C-peptide was measured by an immunoradiometric assay (IM3639, Beckman Coulter, Brea, California, USA). The limit of quantitation was 3.8 pmol/L, and interassay coefficient of variation (c.v.) was 9.1% at 6.5 pmol/L.

### Statistical analysis

Descriptive continuous data were expressed as mean with SD (±SD) or as median and IQR for normally distributed and non-normally distributed data, respectively. Categorical data were presented as number and percentage (%). Baseline characteristics were compared with the χ^2^ test for categorical data, and t-test or Mann-Whitney U test for continuous variables, depending on their distribution.

Logistic regression analysis was used to test the association between the presence of IAH and individual parameters, with stepwise forward selection with entry testing based on the significance of the score statistic, and removal testing based on the probability of the Wald statistic. Log transformation was done for variables not-normally distributed. Participants with fasting C-peptide <300 pmol/L were included in the models, as individuals with higher C-peptide concentrations may not have typical type 1 diabetes. Alcohol, also an expected risk factor for IAH,^27^ was not included in the model due to incomplete data. eGFR was taken into account in the multivariable models because C-peptide is subject to renal excretion.^28^ Three models were evaluated based on (1) Log-transformed C-peptide; (2) presence or absence of C-peptide (< or ≥3.8 pmol/L); (3) categorical C-peptide, defined as either not detectable, 3.8–20 pmol/L and >20 pmol/L concentration. As a sensitivity analysis, we repeated these analyses with exclusion of the 27 participants who were not completely fasted at blood draw. We also analyzed the contribution of variables to the presence of IAH based on the model of Holstein *et al*, excluding the KCNJ11 polymorphisms.^22^ For all analyses, a significance level of p<0.05 was used. Statistical analyses were performed using SPSS (IBM. Released 2015. IBM SPSS Statistics for Windows, V.23.0. IBM).

## Results

Complete data for evaluation were available in 509 individuals with type 1 diabetes. Baseline characteristics of the participants stratified by level of IAH are presented in tables 1 and 2.

**Table 1:** Characteristics of the participants

	Impaired awareness of hypoglycemia		P value
	Absent	Present	
	n=434	n=75	
Age, years	28 (22–51)	49 (31–59)	<0.001
Female gender, n (%)	256 (59.0)	46 (61.3)	0.702
Diabetes duration, years	17 (12–29)	25 (15–39)	<0.001
Diabetes duration >35 years, n (%)	68 (15.7)	22 (29.3)	0.004
Age at diabetes onset, years	12 (8–19)	17 (11–27)	0.001
Age at diabetes onset >18, years, n (%)	120 (27.6)	35 (46.7)	0.001
Body mass index, kg/m^2^	25.0 (22.8–27.6)	25.6 (24.1–29.3)	0.007
Insulin pump use, n (%)	279 (64.3)	45 (60.0)	0.476
Insulin dose (U/day)	55±22	50±21	0.085
Glucose sensor use, n (%)	88 (20.3)	32 (42.7)	<0.001
rtCGM/isCGM (n)	68/20	30/2	<0.001
Alcohol use, n (%)*	123 (35.3)	15 (29.4)	0.405
HbA_1c_, %	7.7 (1.1)	7.7 (1.0)	0.994
HbA_1c_, mmol/mol	61 (12)	61 (11)	0.995
C-peptide continuous, pmol/L	0.0 (0.0–4.5)	0.0 (0.0–0.0)	0.135
C-peptide categorical, <3.8, 3.8–20, >20 pmol/L, n (%)	319/55/60 (73.5/12.7/13.8)	61/7/7 (81.4/9.3/9.3)	0.352
eGFR, mL/min/1.73 m^2^	105±21	95±23	<0.001
Total cholesterol, mmol/L	4.5 (4.0–5.0)	4.5 (4.1–5.2)	0.463
HDL cholesterol, mmol/L	1.5 (1.3–2.0)	1.7 (1.4–2.0)	0.104
LDL cholesterol, mmol/L	2.5 (2.2–3.0)	2.5 (2.2–3.1)	0.820
Triglycerides, mmol/L	0.8 (0.6–1.2)	0.9 (0.7–1.2)	0.201
Albumin:creatinine ratio, mg/mmol	0.4 (0.0–1.1)	0.4 (0.0–1.2)	0.843

Data are presented as mean±SD, median (IQR) or n (%).

*Only available in 399 participants.

eGFR, estimated glomerular filtration rate; HbA_1c_, glycated hemoglobin; HDL, high-density lipoprotein; isCGM, intermittently scanned (flash) glucose monitoring; LDL, low-density lipoprotein; rtCGM, real-time continuous glucose monitoring.

**Table 2:** Complications and medication use in the participants

	Impaired awareness of hypoglycemia		P value
	Absent	Present	
	n=434	n=75	
Self-reported severe hypoglycemia in the past 12 months, n (%)	136 (31.3)	58 (77.3)	<0.001
Self-reported severe hypoglycemia requiring medical intervention, n (%)	19 (4.4)	22 (29.3)	<0.001
Hypertension, n (%)	59 (13.6)	20 (26.7)	0.004
Any microvascular complication, n (%)	198 (45.6)	51 (68.0)	<0.001
Microalbuminuria, n (%)	66 (15.6)	15 (20.0)	0.346
Macroalbuminuria, n (%)	9 (2.1)	0 (0.0)	0.202
Retinopathy, n (%)	113 (26.0)	27 (36.5)	0.063
Neuropathy self-reported, n (%)	106 (24.4)	27 (36.0)	0.035
Macrovascular complications, n (%)	20 (4.6)	9 (12.0)	0.011
Angina pectoris, n (%)	6 (1.4)	0 (0.0)	0.306
MI, n (%)	7 (1.6)	5 (6.7)	0.008
PTCA, n (%)	6 (1.4)	5 (6.4)	0.004
CABG, n (%)	5 (1.2)	4 (5.3)	0.011
TIA/CVA, n (%)	7 (1.6)	3 (4.0)	0.169
Antihypertensive drugs, n (%)	90 (20.7)	27 (36.0)	0.004
Beta-blockers, n (%)	20 (4.6)	7 (9.3)	0.092
Diuretics, n (%)	27 (6.2)	14 (18.7)	<0.001
Calcium antagonists, n (%)	20 (4.6)	5 (6.7)	0.446
RAAS blockers, n (%)	71 (16.4)	20 (26.7)	0.031
Cholesterol-lowering drugs, n (%)	95 (21.9)	28 (37.3)	0.004
Antiplatelet agents, n (%)	23 (5.3)	9 (12.0)	0.027
Antidepressants, n (%)	21 (4.8)	8 (10.7)	0.044

Data are presented as mean±SD, median (IQR) or n (%).

CABG, coronary artery bypass grafting; CVA, cerebrovascular accident; MI, myocardial infarction; PTCA, percutaneous transluminal coronary angioplasty; RAAS, renin-angiotensin-aldosterone system; TIA, transient ischemic attack.

**Figure 1:**
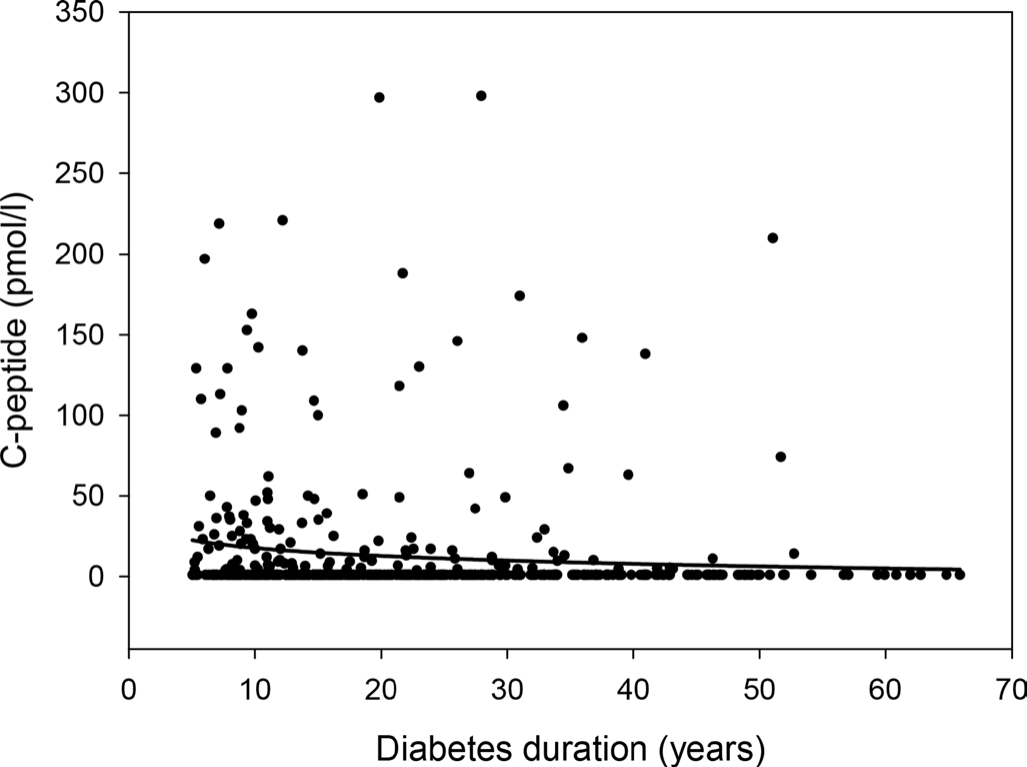
Relationship between C-peptide and diabetes duration in 509 individuals with type 1 diabetes. Regression line shows logarithmic fit.

The median age of the study cohort (IQR) was 32 (23–53) years, 302 (59%) individuals were female, the diabetes duration was 19 (12–30) years and age at diabetes onset was 12 (8–20) years. Furthermore, 249 (49%) individuals had at least one microvascular complication and 29 (6%) individuals had a macrovascular complication. In total, 324 (64%) participants administered insulin by continuous subcutaneous insulin infusion using a pump. Mean HbA_1c_ concentration (SD) was 60 (12) mmol/mol/7.7 (1.1)%. In 129 (25%) individuals, residual C-peptide secretion was detected; median C-peptide concentration among all participants was 0.0 (0.0–3.9) pmol/L, 62 (12.2%) had C-peptide concentrations between 3.8 and 20 pmol/L, and 67 (13.2%) had C-peptide concentrations >20 pmol/L. C-peptide concentration decreased with longer duration of diabetes (figure 1).

Severe hypoglycemia in the past 12 months was reported by 196 participants (38.5%), 41 (8.1%) of whom required medical intervention. The prevalence of severe hypoglycemia was similar across sex and not related to poorer glycemic control, longer diabetes duration, the presence of microvascular or macrovascular complications, or use of beta-blockers or antidepressants. However, prevalence of severe hypoglycemia was lower in people with demonstrable C-peptide versus those with absent C-peptide (30% vs 41%, p=0.025).

**Figure 2:**
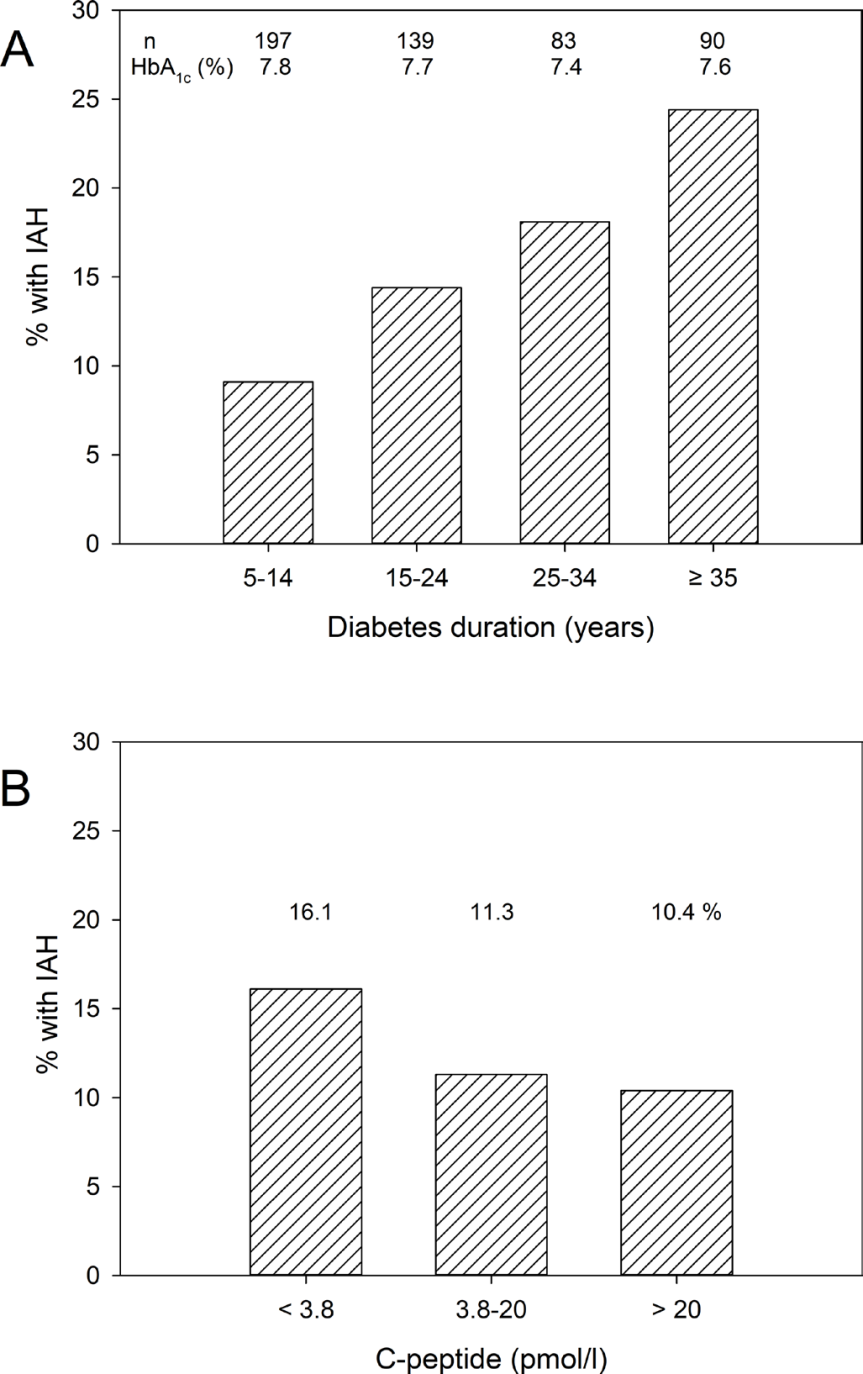
(A) Prevalence of impaired awareness of hypoglycemia (IAH) in relation to diabetes duration and HbA1c concentrations. Overall p for % with IAH by χ^2^=0.006. (B) Prevalence of IAH in relation to serum C-peptide levels. Overall p for % with IAH by χ^2^=0.352.

The median value of the modified Clarke score was 1.0 (0.0–2.0). In total, 75 (14.7%) individuals had a modified Clarke score of ≥3 and were classified as having IAH. As demonstrated in table 1, participants with IAH were older, had longer diabetes duration, a higher age of diabetes onset, and significantly more often hypertension, microvascular complications and macrovascular complications. Figure 2A shows the relationship between longer diabetes duration and higher prevalence of IAH, which amounted to 25% in those with diabetes duration >35 years, while figure 2B shows the uncorrected prevalence of IAH in relation to serum C-peptide levels. People with IAH were significantly more commonly treated with antihypertensive drugs, cholesterol-lowering drugs and antiplatelet agents compared with people without IAH (table 2). There was no significant difference in HbA_1c_ and C-peptide concentrations between people with and without IAH. Among people with IAH, 58 (77.3%) reported at least one episode of severe hypoglycemia in the preceding year, and 22 (29.3%) had a severe hypoglycemic incident necessitating medical intervention (table 2). Thus, IAH was associated with a nine times higher risk (OR 9, 1, 95% CI 4.6 to 17.8, p<0.001) of having a severe hypoglycemia necessitating medical intervention in the preceding year.

In the multivariable logistic regression models, higher C-peptide was significantly associated with the absence of IAH, while especially age at onset, BMI and presence of microvascular complications showed a positive association with the presence of IAH (table 3 and online supplemental tables 1 and 2). The sensitivity analyses, in those who were fasting at blood draw, yielded similar results: model 1, log C-peptide OR 0.63 (0.40 to 0.98), p=0.040; model 2, C-peptide dichotomous OR 0.51 (0.26 to 0.98), p=0.042). We reproduced the analysis published by Holstein *et al* in 2005 (online supplemental table 3)^22^ and show that residual C-peptide secretion was negatively associated with IAH, when assessed in this model that also included age, HbA_1c_, and short and long duration of diabetes.

10.1136/bmjdrc-2021-002288.supp1 Supplementary data

10.1136/bmjdrc-2021-002288.supp2 Supplementary data

10.1136/bmjdrc-2021-002288.supp3 Supplementary data

**Table 3:** Multivariable association of impaired awareness of hypoglycemia with clinical parameters, C-peptide continuous log transformed

	Full model			Forward stepwise model		
	OR	95% CI	P value	OR	95% CI	P value
Diabetes duration	1.02	(0.99 to 1.04)	0.261			
Log age at onset of diabetes, years	4.01	(1.63 to 9.86)	0.003	3.99	(1.77 to 9.01)	0.001
BMI, kg/m^2^	1.07	(1.02 to 1.13)	0.011	1.08	(1.03 to 1.14)	0.003
Hypertension	1.20	(0.63 to 2.32)	0.576			
Microvascular complications	1.83	(1.00 to 3.33)	0.048	2.28	(1.34 to 3.90)	0.003
Macrovascular complications	1.52	(0.56 to 4.18)	0.414			
Log C-peptide, pmol/L	0.66	(0.42 to 1.02)	0.062	0.63	(0.40 to 0.98)	0.040
eGFR (CKD-EPI), mL/min/1.73 m^2^	1.00	(0.99 to 1.02)	0.954			
Beta-blocker use	0.74	(0.26 to 2.17)	0.589			

BMI, body mass index; CKD-EPI, Chronic Kidney Disease Epidemiology Collaboration; eGFR, estimated glomerular filtration rate.

## Discussion

In this cross-sectional study among 509 individuals with type 1 diabetes, the overall prevalence of IAH was 15%. Longer duration of diabetes, higher age, older age at diabetes onset, higher body mass index and lower eGFR were associated with presence of IAH, as well as the presence of microvascular and macrovascular complications, and a severe hypoglycemia in the preceding year. In multivariable analyses, the presence of residual C-peptide secretion was negatively associated with a prevalence of IAH, while the presence of microvascular complications, higher BMI and older age at onset were positively associated with the presence of IAH. These data confirm the results of previous studies and indicate that even in these modern times, IAH can be a major consequence of long-standing diabetes.

Two previous studies addressed the relationship of C-peptide and IAH. In a retrospective cohort study among 167 persons with type 1 diabetes, no difference could be demonstrated in the presence of IAH, measured using the Gold questionnaire, between groups with (defined as 10–200 pmol/L) and without (<10 pmol/L) C-peptide secretion.^19^ However, flash glucose monitoring data (Freestyle Libre, Abbott, Witney, UK) showed that time below range and number of low-glucose events were lower in individuals with preserved C-peptide, and low-C-peptide individuals were more likely to report not always being aware of hypoglycemia.^19^ Although the Clarke and the Gold questionnaires have good concordance,^29^ differences in instruments use (ie, questionnaires and glucose measurement techniques) should be considered when comparing the results of studies on IAH. It should also be noted that Gibb *et al* used random and not fasting C-peptide measurements in their statistical analyses.^19^ Some people with type 1 diabetes who are considered secretors of C-peptide may have a definite increase of C-peptide after a mixed meal.^30^ Holstein *et al* demonstrated in a cohort of 217 individuals that the absence of C-peptide was independently associated with IAH. However, their statistical model was confined to the presence or absence of C-peptide, age, HbA_1c_, long versus short duration of diabetes, and KCNJ11 polymorphisms.^22^ We were able to replicate their findings by assessing the same parameters in the model, although we could not evaluate the association with the described KCNJ11 polymorphisms.

The prevalence of IAH (15%) in our study employing the validated Dutch version of the Clarke questionnaire^25^ is lower compared with previous studies. In adults with type 1 diabetes, a prevalence of 19% was reported using the Gold questionnaire,^7^ while in an online survey, 23% of 418 participants reported impaired awareness, while 15% had uncertain awareness.^31^ As the incidence of IAH increases with age and diabetes duration,^3 4 27^ the lower prevalence of IAH in our study may be explained by our relatively young study population (median age of 32 years) as compared with the mean age of 48 years in the study of Geddes *et al*.^7^ The relation between older age and longer diabetes duration and IAH is also confirmed by our findings, and 25% of those with >35 years duration of diabetes reported IAH.

It has to be emphasized that actual hypoglycemia rates may influence the association of C-peptide with the presence of IAH. Frequent hypoglycemia or earlier severe hypoglycemia is an important risk factor for IAH.^5^ Indeed, there was a strong association between presence of IAH and having a severe hypoglycemia necessitating medical intervention in the preceding year. Furthermore, persistent C-peptide secretion and thus preserved endogenous insulin production is only associated with reduced hypoglycemia and not HbA_1c_.^18^ This could indicate that C-peptide influences only hypoglycemia and not long-term glycemic outcome measured by HbA_1c_, suggesting added value to include hypoglycemia rates into the analysis. However, it is possible that there is another pathophysiologic mechanism underlying the association between C-peptide and hypoglycemia versus C-peptide and IAH. Furthermore, presence of C-peptide and the levels of glucose also depend on the intensity of insulin treatment.^16 17^ It could therefore be argued that the amount of daily insulin units should also have been included in the regression models. However, this is a parameter that is prone to considerable variation depending on, for instance, nutritional factors and differences in physical activity.

As opposed to expected, age at onset was positively (and not negatively) associated with the presence of IAH. In people with type 1 diabetes onset at an early age, autoimmune response against beta-cells is fiercer and persistent C-peptide secretion is less often present. As such, younger age at diabetes onset results in an increased risk of diabetes-related complications.^18 32 33^ It may be suggested that the presence of IAH is also associated with younger age at diabetes onset. However, in previous research, the probability of developing retinopathy was lower when type 1 diabetes was diagnosed before the age of 5 compared with the age groups 5–11 and >11 years. This could also apply to IAH.^34^ Duration of diabetes was no longer associated with the presence of IAH in the multivariable model, as longer diabetes duration was also associated with higher prevalence of microvascular complication, lower C-peptide levels and higher BMI, other factors associated with IAH presence (table 3).

In our study, beta-blocker use was not associated with IAH, as demonstrated by others.^27^ This may be explained by our relatively young study population and limited use of this type of medication. Beta-blocker use has been associated with increased rate of severe hypoglycemia in adults with diabetes.^35^ Especially in people with IAH, beta-blockers may less often be prescribed because clinicians may fear the effect they can have on attenuating the symptoms of hypoglycemia.^2^ However, a recent study suggested that beta-blocker use was not related to hypoglycemia unawareness, or burden in hospitalized high-risk insulin-requiring people with diabetes.^36^ The use of a glucose sensor was higher in participants with IAH. It should be taken into consideration that in The Netherlands individuals with type 1 diabetes and IAH are eligible for both the prescription and the reimbursement of real-time glucose monitoring. Therefore, we consider the use of a glucose sensor a consequence of and not a risk factor for IAH, and did not include this parameter in the multivariable analyses. Finally, BMI was positively associated with the presence of IAH. No previous data on the association of BMI and IAH are available. It can be hypothesized that frequent hypoglycemia may lead to increased caloric intake and thereby higher BMI, as may be regular preventive ‘snacking’ to prevent hypoglycemia. Additionally, a strong impediment for physical exercise in people with type 1 diabetes is fear of severe hypoglycemia,^37^ a common adverse event of physical exercise in individuals with type 1 diabetes,^38^ which may also be a factor contributing to higher BMI.

In the present study, we did not observe any difference in HbA_1c_ levels between people with and without IAH. Glycemic impact, displayed by HbA_1c_, may be a risk factor for the presence of IAH. On one hand, individuals with IAH may have lower HbA_1c_ levels compared with those without IAH because of the fact that tighter glycemic control is associated with more frequent hypoglycemic episodes and loss of awareness as a consequence.^39^ On the other hand, those with IAH—and their caregivers—may aim to achieve slightly higher HbA_1c_ levels in order to avoid hypoglycemia and therefore more often are hyperglycemic, resulting in an increased risk for developing diabetes-related complications.^14 40^ Unfortunately, the current study lacks long-term HbA_1c_ measurements to evaluate this further.

### Study limitations

Strengths of this study include the multicenter design, large sample size and considerable phenotyping of the study population. A limitation of this study is that no conclusion can be drawn concerning causality of our findings due to its cross-sectional design. A number of variables like alcohol consumption had missing data, which could have influenced the accuracy of its association with IAH. Finally, it can be hypothesized that some people with IAH are unaware of the fact that they do not notice hypoglycemic episodes and therefore under-report hypoglycemia using questionnaires, as such continuous glucose measurements using a sensor would have been of added value.

## Conclusions

This study demonstrates that residual C-peptide secretion is associated with lower prevalence of IAH in people with type 1 diabetes. Furthermore, we demonstrated that higher age at onset, presence of microvascular complications and higher BMI were independently associated with the presence of IAH.

## Data Availability

Data are available upon reasonable request. Data are available upon reasonable request. All data requests will be subject to relevant GDPR and ethics considerations.
