# Supplementary material for: Residual C-peptide secretion and hypoglycemia awareness in people with type 1 diabetes
Source: BMJ Open Diabetes Res Care. 2021 Sep 15;9(1):e002288. doi: 10.1136/bmjdrc-2021-002288 (PMC8444236; doi:10.1136/bmjdrc-2021-002288)
Supplement: Supplementary data [file bmjdrc-2021-002288supp001.pdf]

Supporting information

Table S1. Multivariable association of impaired awareness of hypoglycaemia with clinical parameters, C-peptide dichotomous

	Full model			Forward stepwise model		
	OR	95%CI	*P*	OR	95%CI	*P*
Diabetes duration	1.02	[0.99-1.04]	0.253			
Log Age at onset of diabetes, years	3.98	[1.62-9.80]	0.003	3.95	[1.75-8.93]	0.001
BMI kg/m^2	1.07	[1.02-1.13]	0.012	1.08	[1.02-1.14]	0.004
Hypertension	1.19	[0.62-2.28]	0.609			
Microvascular complications	1.81	[1.00-3.30]	0.051	2.26	[1.32-3.87]	0.003
Macrovascular complications	1.49	[0.54-4.08]	0.436			
C-peptide, dichotomous	0.54	[0.28-1.06]	0.073	0.51	[0.26-0.98]	0.042
eGFR (CKD-EPI), ml/min/1.73m^2	1.00	[0.99-1.02]	0.945			
Beta-blocker use	0.76	[0.26-2.21]	0.614			
