# Supplementary material for: Residual C-peptide secretion and hypoglycemia awareness in people with type 1 diabetes
Source: BMJ Open Diabetes Res Care. 2021 Sep 15;9(1):e002288. doi: 10.1136/bmjdrc-2021-002288 (PMC8444236; doi:10.1136/bmjdrc-2021-002288)
Supplement: Supplementary data [file bmjdrc-2021-002288supp002.pdf]

Supporting information

Table S2. Multivariable association of impaired awareness of hypoglycaemia with clinical parameters, C-peptide categorical

	Full model			Forward stepwise model		
	OR	95%CI	*P*	OR	95%CI	*P*
Diabetes duration	1.01	[0.99-1.04]	0.266			
Log Age at onset of diabetes, years	4.00	[1.63-9.86]	0.003	3.21	[1.48-6.96]	0.003
BMI kg/m^2	1.07	[1.02-1.13]	0.011	1.08	[1.02-1.14]	0.004
Hypertension	1.19	[0.62-2.28]	0.604			
Microvascular complications	1.82	[1.00-3.31]	0.050	2.35	[1.38-4.01]	0.002
Macrovascular complications	1.51	[0.55-4.15]	0.423			
C-peptide 3.8-20 pmol/.l	0.63	[0.26-1.51]	0.300			
C-peptide > 20 pmol/l	0.47	[0.20-1.15]	0.099			
eGFR (CKD-EPI), ml/min/1.73m^2	1.00	[0.99-1.02]	0.959			
Beta-blocker use	0.75	[0.26-2.18]	0.595			
