# Supplementary material for: Residual C-peptide secretion and hypoglycemia awareness in people with type 1 diabetes
Source: BMJ Open Diabetes Res Care. 2021 Sep 15;9(1):e002288. doi: 10.1136/bmjdrc-2021-002288 (PMC8444236; doi:10.1136/bmjdrc-2021-002288)
Supplement: Supplementary data [file bmjdrc-2021-002288supp003.pdf]

Supporting information

Table S3. Multivariable analysis applied to our dataset according to the methodology of Holstein et al. [22]

	OR	95%CI	*P*
C-peptide (negative vs positive)	2.49	[1.08-5.71]	0.032
HbA1c (%)	1.20	[0.90-1.61]	0.218
Diabetes duration (>20 yrs vs <10 yrs)	1.07	[0.40-2.85]	0.888
Age (yrs)	1.02	[1.00-1.04]	0.043
